# Case Report: Biventricular Noncompaction Cardiomyopathy With Pulmonary Stenosis and Bradycardia in a Fetus With KCNH2 Mutation

**DOI:** 10.3389/fgene.2022.821226

**Published:** 2022-02-24

**Authors:** Hairui Sun, Xiaowei Liu, Xiaoyan Hao, Xiaoxue Zhou, Jingyi Wang, Jiancheng Han, Mengmeng Liang, Hongjia Zhang, Yihua He

**Affiliations:** ^1^ Department of Echocardiography, Beijing Anzhen Hospital, Capital Medical University, Beijing, China; ^2^ Cipher Gene LLC, Beijing, China; ^3^ Department of Cardiac Surgery, Beijing Anzhen Hospital, Capital Medical University, Beijing, China

**Keywords:** left ventricular noncompaction, long QT syndrome, congenital heart disease, prenatal diagnosis, KCNH2 gene mutation

## Abstract

**Background:** Left ventricular noncompaction (LVNC) is a rare cardiomyopathy, long QT syndrome (LQTS) is a rare ion channel disease, and simultaneous occurrence of both is even rarer. Further clinical reports and studies are needed to identify the association between LVNC and LQTS and the underlying mechanism.

**Methods and Results:** A 26-year-old primigravida was referred at 25 weeks gestation for prenatal echocardiography due to fetal bradycardia detected during the routine ultrasound examination. The echocardiographic findings were consistent with biventricular noncompaction cardiomyopathy (BVNC) with pulmonary stenosis and suspected LQTS. After detailed counseling, the couple decided to terminate the pregnancy, and subsequent postmortem examination confirmed BVNC and pulmonary stenosis. Then, A trio (fetus and the parents) whole-exome sequencing (WES) and copy number variation sequencing (CNV-seq) were performed. CNV-seq identified no aneuploidy or pathogenic CNV. A *de novo* missense variant in KCNH2 (NM_000238.3:c.1847A > G,p.Tyr616Cys) was identified by WES. This KCNH2 missense mutation was classified as pathogenic according to the American College of Medical Genetics and Genomics and the Association for Molecular Pathology variant interpretation guidelines.

**Conclusion:** We report the first prenatal case of KCNH2 mutation presenting with LVNC combined with bradycardia and second-degree 2:1 atrioventricular block. Importantly, this case reminds clinicians to systematically search ion channel gene mutations in patients with LVNC and arrhythmia.

## Introduction

Left ventricular noncompaction (LVNC) is rare genetic cardiomyopathy (Towbin et al., 2015). Genes associated with LVNC usually include those encoding sarcomere, ion channels, nuclear envelope, and chaperone proteins. Many ion-channel genes, such as SCN5A, RYR2, KCNQ1, and HCN4, have been associated with LVNC, but the underlying molecular mechanisms are unknown (Milano et al., 2014; Nakashima et al., 2013; Schweizer et al., 2014; Towbin, 2014). KCNH2, and an ion-channel gene, encodes the pore-forming subunit of a rapidly activating-delayed rectifier potassium channel that plays a critical role in the final repolarization of the ventricular action potential (Gianulis and Trudeau, 2011). Mutations in the KCNH2 gene cause long QT syndrome type 2 (LQTS2, MIM:613688) (Amin et al., 2008). The combination of LVNC with LQTS is scarce, and clinical reports of KCNH2 variants in such cases are even rarer. Due to the scarcity of clinical reports, LVNC has not been recognized as a feature of LQTS2. Here we report the first fetal case, to our knowledge, with KCNH2 mutation presenting with LVNC, LQTS, and sinus bradycardia. We also reviewed the literature to identify additional cases of KCNH2 mutation with LVNC-LQTS combined phenotype.

## Materials and Methods

### Editorial Policies and Ethical Considerations

This study was approved by the Ethics Committee of Beijing Anzhen Hospital, Capital Medical University and adhered to the tenets of the Declaration of Helsinki. Informed written consent was obtained from the parents of the fetus.

### Fetal Ultrasound and Echocardiography Examination

A complete fetal echocardiographic examination, including twodimensional (2D), M-mode, color, and pulse Doppler echocardiography, was performed using the General Electric Voluson E8 ultrasound system with transabdominal 2- to 4- MHz curvilinear transducers (GE Healthcare Ultrasound, Milwaukee, WI, United States) according to the American Society of Echocardiography guidelines and standards for performance of the fetal echocardiogram (Rychik et al., 2004).

### Copy Number Variation Sequencing (CNV-Seq) and Whole-Exome Sequencing (WES)

Both CNV-seq and WES were done in the setting of a purely research-based protocol, and performed using methods as previously described on genomic DNA from the deceased fetus and the parents (Sun et al., 2019). Briefly, genomic DNA was extracted, hybridized and enriched for whole-exome sequencing. The captured libraries were sequenced using Illumina NovaSeq 6,000 (Illumina, Inc., San Diego, CA, United States). Then, the sequencing data were aligned to the human reference genome (hg38/GRCh38) using BWA (http://bio-bwa.sourceforge.net/) and PCR duplicates were removed by using Picard v1.57 (http://picard.sourceforge.net/). GATK (https://software.broadinstitute.org/gatk/) was applied for variant calling. ANNOVAR (http://wannovar.wglab.org/) was used for variant annotation and interpretation. We determined the frequency of each variant in the dbSNP150 (https://www.ncbi.nlm.nih.gov/snp/), 1,000 Genomes Project (http://www.internationalgenome.org/) and gnomAD (https://gnomad.broadinstitute.org/) to remove common SNPs (minor allele frequency >0.1%). Then, non-synonymous, splicing, frameshift and non-frameshift variants, as well as variants located in splice sites within 20 base pairs of an exon, were prioritized for evaluation. SIFT (http://sift.jcvi.org), PolyPhen-2 (http://genetics.bwh. harvard. edu/pph2), MutationTaster (http://www.mutation taster.org) and CADD (http://cadd.gs.washington.edu) were used to predict the pathogenicity of missense variants, while HSF (http://www.umd.be/HSF3/), and MatEntScan (Yeo and Burge, 2004) were used to evaluate the effects on splicing. Missense variants not presenting damaging results in any protein function prediction from SIFT, Polyphen2, MutationTaster, and CADD were excluded. Intronic variants not presenting damaging results in any prediction from HSF and MatEntScan were excluded. Pathogenicity of variants was determined according to current ACMG guidelines that recommend classifying variants into five categories: pathogenic, likely pathogenic, uncertain significance, likely benign or benign (Richards et al., 2015). Sanger sequencing was used to validate the presence of positive genetic results.

## Results

### Clinical Phenotypes

A 26-year-old primigravida was referred at 25 weeks’ gestation for prenatal echocardiography due to fetal bradycardia detected during the routine ultrasound examination. The woman was healthy with no significant family history and did not take any medication. Her anti-Ro/SSA and anti-La/SSB antibody status were both negative. She and her partner were non-consanguineous.

**FIGURE 1:**
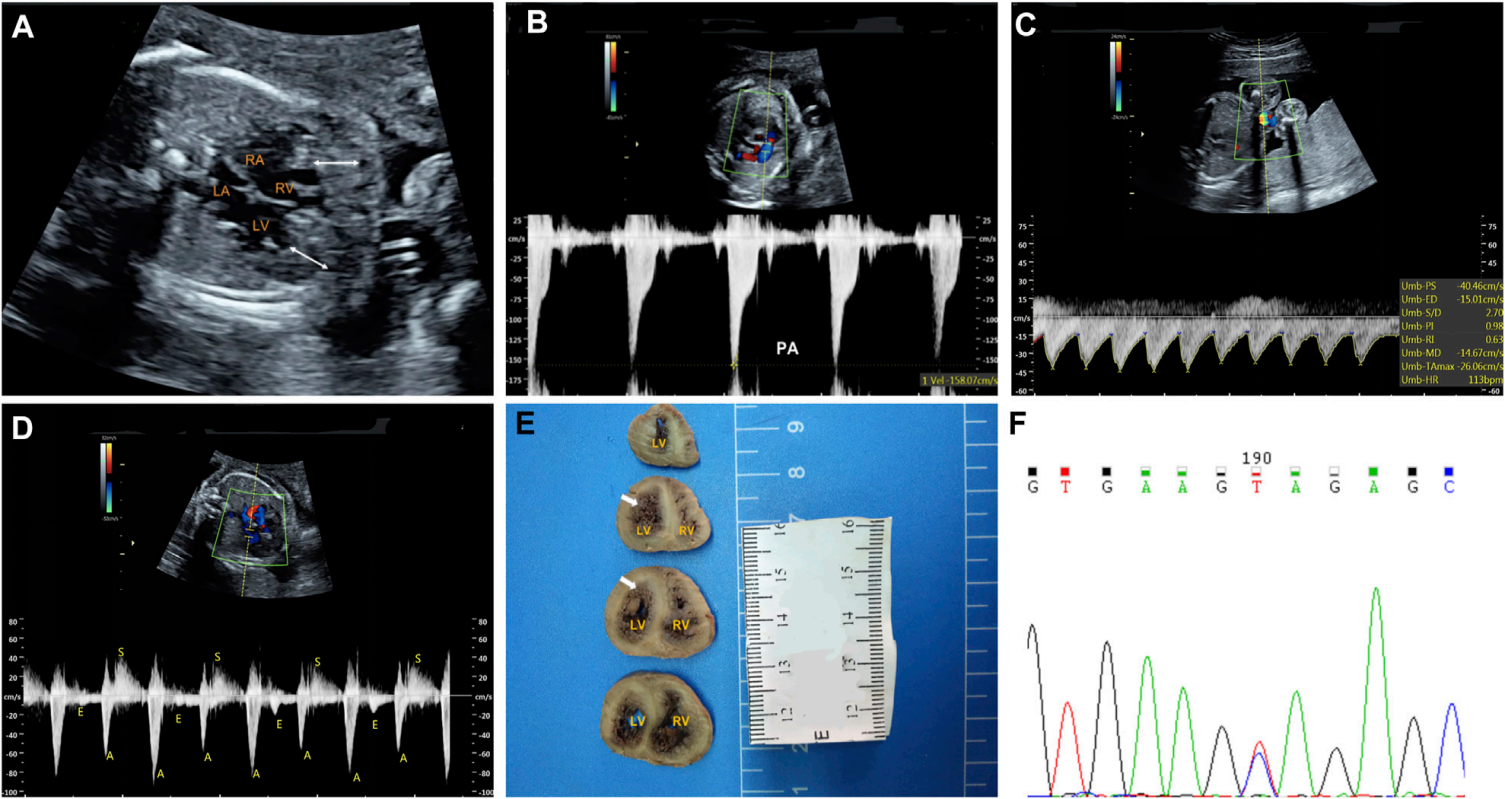
Clinical phenotypes and molecular findings of the fetus. (**A–D)**: Echocardiography of the fetus at 25 weeks’ gestation. The phenotype includes biventricular noncompaction cardiomyopathy **(A)**, pulmonary stenosis **(B)**, sinus bradycardia **(C)** and second-degree 2:1 atrioventricular block (D). A. The area between the white arrows indicate numerous ventricular trabeculae **(C)**. Sinus bradycardia was seen with ventricular rate of 113 bpm by umbilical artery blood flow spectrum **(D)**. Mitral inflow and left ventricular outflow spectrum shows a 2:1 second-degree atrioventricular block **(E)**: Pathological anatomy shows that the noncompaction myocardium below the level of the left ventricular papillary muscle is obvious. The white arrows indicate the noncompaction myocardium **(F)**: Sanger sequencing shows that the mutation is heterozygous in the fetus. LA: left atrium; LV: left ventricle; RA: right atrium; RV: right ventricle.

The fetal echocardiography identified noncompacted layers in both ventricles. An extensive trabeculated layer, with multiple deep intratrabecular recesses filled with blood directly from the ventricular cavity, was seen (Figure 1A). Moreover, a thickened, stenotic pulmonary valve was noted with a peak velocity of 158 cm/s shown by spectral Doppler (Figure 1B). There was fetal bradycardia; second-degree 2:1 atrioventricular block was seen by spectral Doppler and M-mode Echocardiography (Figures 1C,D). The fetal echocardiographic findings were consistent with biventricular noncompaction cardiomyopathy (BVNC) with pulmonary stenosis, second-degree 2:1 atrioventricular block, and sinus bradycardia.

At the time of the fetal diagnosis, the family was counseled about the potential overall poor prognosis for this fetus related to the BVNC with the second-degree atrioventricular block and pulmonary stenosis. Finally, the family decided to terminate the pregnancy and undergo genetic testing. The pregnancy was terminated at 26 weeks’ gestation. Subsequent postmortem examination confirmed BVNC and pulmonary stenosis (Figure 1E).

### Molecular Findings

A trio (fetus and the parents) CNV-seq and WES were performed to determine the underlying genetic cause of the fetal cardiac phenotype. CNV-seq analysis identified no chromosomal abnormalities. The WES analysis initially identified 83,186 initial variants. The filtering cascades for WES data are listed in Supplementary Table S1. After four filters of the variants data for WES data, 91 variants were kept (Supplementary Table S2). Finally, we identified a *de novo* missense variant in KCNH2 (NM_000238.3:c.1847A > G,p.Tyr616Cys) in the fetus (Figure 1F), while no pathogenic variants in other known genes associated with cardiomyopathy or arrhythmias were identified. This KCHN2 variant was not found in the biggest general population database (gnomAD, https://gnomad.broadinstitute.org) and in-house control database and showed a deleterious effect by multiple in silico algorithms. The variant has been reported previously in several individuals with LQTS (Kapplinger et al., 2009; Ware et al., 2012), and ClinVar database (http://www.ncbi.nlm.nih.gov/clinvar) contains an entry for this variant (Variation ID: 67295). In addition, the Tyr616 is located in the intramembrane pore-forming H5 domain of KCNH2, and mutations at surrounding codons (Leu615Phe, Leu615Val, Ala614Val, and Thr613Met) have also been reported in association with LQTS, supporting the functional importance of this region of the protein. Furthermore, functional studies carried out by Anderson et al. (2014) demonstrate Tyr616Cys generated minimal current, suggesting altered channel permeability as a mechanism that leads to Prolonged QT interval. In conclusion, the variant is classified as pathogenic according to the 2015 American College of Medical Genetics and Genomics guidelines (Richards et al., 2015).

## Discussion

In this report we present the first fetal case with KCNH2 mutation presenting with BVNC, pulmonary stenosis, second-degree 2:1 atrioventricular block, and sinus bradycardia. LVNC is an increasingly recognized type of cardiomyopathy characterized by excessive trabeculation of the ventricles with deep intertrabecular recesses. While LVNC was classified as distinct cardiomyopathy by the American Heart Association (Maron et al., 2006), the European Society of Cardiology categorizes it as unclassified cardiomyopathy (Elliott et al., 2007). In the early fetal period, about 12 weeks, the myocardium is widely formed by trabeculae. These trabeculations undergo a compaction process, mainly finished before the 16–18 weeks of pregnancy (Faber et al., 2021; Finsterer et al., 2017). According to the non-compaction theory, LVNC results from the arrest of endomyocardial morphogenesis, leading to trabecular compaction failure (Hussein et al., 2015).

In the absence of a known family history, the diagnosis of fetal LQTS is based on the correct recognize of the signature rhythms, such as second-degree AVB, and sinus bradycardia. Second-degree AVB is the signature rhythm of LQTS in the perinatal period, and have been reported in about 25% of fetal LQTS cases (Horigome et al., 2010; Mitchell et al., 2012). Sinus bradycardia is also a common manifestation of fetal LQTS, and has been reported in as many as 44–66% of fetuses diagnosed with LQTS (Cuneo et al., 2013; Greene et al., 2013; Horigome et al., 2010; Mitchell et al., 2012). In this fetus, a prenatal LQTS was highly suspected based on the following: second-degree AVB(Cuneo et al., 2013; Greene et al., 2013), sinus bradycardia (Cuneo et al., 2013; Greene et al., 2013; Mitchell et al., 2012), and the report of the same KCNH2 in several individuals with LQTS (Kapplinger et al., 2009; Ware et al., 2012). However, we were unable to make a definitive diagnosis because a QT prolongation was not proven.

Through systematic literature review, we identified several additional cases of KCNH2 mutation with LVNC-LQTS combined phenotype (AlSenaidi et al., 2014; Ogawa et al., 2009; Rammes et al., 2017). The first association between LVNC and KCNH2 mutations was described by Ogawa et al. (2009) reporting 2 unrelated individuals with isolated LVNC and LQTS carrying missense mutations in KCNH2. Subsequent AlSenaidi et al. (2014) reported a 5-year-old girl of consanguineous Oman parents carrying a KCNH2 homozygous frameshift mutation in association with phenotypes including LVNC, dilated ascending aorta and LQTS. Interestingly, both the parents of the girl carried this KCNH2 heterozygous mutation but neither presented with LVNC, indicating that LVNC is incomplete in LQTS patients with KCNH2 mutations. Recently, Rammes et al. (2017) also reported a familial case, in which both the proband and her son presented with LVNC, and LQTS carrying a published pathogenic variant in KCNH2. These increasing reports suggest that the coexistence of LVNC and arrhythmia may not be rare, significantly, as the detection rate of LVNC is gradually increasing with the advances of echocardiography and MRI.

Interestingly, KCNH2 mutations have also been reported in patients with isolated LVNC. Recent works of LVNC by the groups of Miszalski-Jamka and Wang have reported several individuals carrying KCNH2 mutations in association with LVNC(Miszalski-Jamka et al., 2017; Wang et al., 2017). Notably, no arrhythmia has been reported in these subjects. In addition, in the study by Wang et al. (2017), the number of rare variants in KCNH2 was significantly enriched in LVNC patients compared with the control group, further supporting the association between LVNC and KCNH2 mutation. In several recent independent LVNC cohorts, the variation burden of ion channel genes were as high as 8.8–14.7 (Hirono et al., 2020; Cambon-Viala et al., 2021; Miszalski-Jamka et al., 2017; Wang et al., 2017), significantly higher than in the general population. This suggests that ion-channel dysfunction may play a role in the pathogenesis of LVNC(Milano et al., 2014; Nakashima et al., 2013; Shan et al., 2008; Towbin, 2014). Although the exact mechanism is unclear, several hypotheses have been proposed. They suggest that direct protein-protein interaction between the ion channel gene products and the sarcomere may induce ventricular noncompaction or that ventricular noncompaction is an acquired adaptive remodeling feature in response to impaired conduction (Ergul et al., 2011; Brescia et al., 2013; Caliskan et al., 2012; Steffel and Duru, 2011; Zhang et al., 2013).

## Conclusion

In summary, we present the first fetus with LVNC-LQTS combined phenotype and KCNH2 mutation. This case reminds clinicians that ion channel gene variants should be searched systematically in LVNC patients, especially in the arrhythmia phenotype.

## Data Availability

The datasets for this article are not publicly available due to concerns regarding participant/patient anonymity. Requests to access the datasets should be directed to the corresponding author.
